# Selenium nanoparticles modified niobium MXene for non-enzymatic detection of glucose

**DOI:** 10.1038/s41598-025-85748-y

**Published:** 2025-01-11

**Authors:** Prabisha K. E., Neena P. K., Menon Ankitha, P. Abdul Rasheed, P. V. Suneesh, T. G. Satheesh Babu

**Affiliations:** 1 Department of Chemistry, Amrita School of Physical Sciences Coimbatore, Amrita Vishwa Vidyapeetham, Coimbatore, 641112 India; 2 Amrita Biosensor Research Laboratory, Amrita School of Engineering Coimbatore, Amrita Vishwa Vidyapeetham, Coimbatore, 641112 India; 3 Department of Chemistry, Indian Institute of Technology, Palakkad, Kerala 678623 India; 4 Department of Biological Sciences and Engineering, Indian Institute of Technology, Palakkad, Kerala 678623 India

**Keywords:** 2D nanomaterials, Nb_2_CT_x_-MXenes, Glucose, Amperometry, Selenium nanoparticles, Diagnostic markers, Sensors

## Abstract

Glucose sensing remains a crucial need as diabetes is a worldwide concern. This work reports the application of Nb_2_CT_*x*_—selenium nanoparticle composite material for the nonenzymatic sensing of glucose. The surface morphology of the synthesized composite was analyzed using various microscopic techniques like scanning electron microscopy, transmission electron microscopy, and its structural properties were analyzed using diffraction and spectroscopic methods. A gold disc electrode was modified using the nanocomposite and tested for glucose in an alkaline medium. The sensing was based on the oxidation of glucose on the catalytic surface. The glucose was quantified amperometrically at a significantly lower overpotential of 0.16 V. The sensor showed a good detection range from 2 to 30 mM with a sensitivity of 4.15 µA mM^−1^ cm^−2^ and a detection limit of 1.1 mM.

**Supplementary Information:**

The online version contains supplementary material available at 10.1038/s41598-025-85748-y.

## Introduction

Biosensors have gained interest from researchers in diverse fields due to the enormous growth of the healthcare, medical and pharmaceutical sectors. Despite the hurdles faced in the development of biosensors, the global biosensing devices market is expected to reach USD 31.5 billion by 2028^1^. 800 million of the global population are diabetic which paves way for the rise of glucose biosensor market which is estimated to be USD 9.68 billion in 2024^2^. The advancement in biosensing technology ranges from simple enzymatic or non-enzymatic sensors to lab-on-chip and hospital-on-chip devices^3^. The rising demand for rapid, and sensitive detection of various biomarkers, and pathogens associated with different physiological conditions, have paved the way for the incorporation of advanced materials 2D materials, and core–shell nanoparticles onto the sensors^4^. The use of these materials has enabled the incorporation of multiple technologies along with the sensor to make multi-module analyzing, a reality.

Nanomaterials have tremendous potential in biosensing application due to their advantages like high surface area, catalytic activity, and selectivity towards biomolecules^5^. Nanomaterials such as metal oxides^6–8^, bimetallic/alloys^9^, carbon nanomaterials^10^, layered double hydroxides^11^ and conducting polymers^12^ were used for electrochemical detection of different biomolecules. Among the various nanomaterials studied, two-dimensional (2D) nanomaterials such as graphene^13^, borophene^14^ and metal–organic frameworks^15^ have gained researcher’s attention due to their large surface area and surface tunability. MXenes are a quickly growing class of 2D materials, which are essentially transition metal carbides, nitrides, and carbonitrides. They have garnered significant attention due to their unique structural and chemical versatility^16^. MXenes follow the general formula M_n+1_X_n_T_*x*_ (where M represents an early transition metal, X is carbon and/or nitrogen, and T_*x*_ represents surface terminations such as O, OH, F, or Cl), and exhibit a diverse range of tunable properties. These properties are derived from the wide variety of possible compositions and surface terminations, which enable MXenes to possess excellent electrical conductivity, highly reactive surfaces, and large surface area^17,18^. Among the wide array of 2D materials, MXenes stand out due to their remarkable properties such as excellent dispersibility in solutions, ease of functionalization, high biocompatibility, and the ability to form durable, stable films. Moreover, MXenes demonstrate exceptional metallic conductivity, with values reaching as high as 20,000 S/cm far exceeding the typical conductivity of materials like graphene, which is around 2000 S/cm^19,20^. These characteristics make MXenes an ideal candidate for sensing applications, where sensitivity, surface interaction, and signal transduction are crucial.

Incorporation of highly active nanomaterials with MXenes is expected to enhance their performance. An ultrasensitive and selective electrochemical sensor for dopamine was developed using Nb_2_CT_*x*_ and MoS_2_ heterostructures^21^. Nb_2_CT_*x*_@MoS_2_ modified carbon cloth was able to detect dopamine over the concentration range of 1 fM to 100 µM with a detection limit of 0.23 fM which is the lowest LOD for dopamine amongst the reported Nb_2_CT_*x*_ based nanocomposites. An electrochemical sensor for the detection of immunosuppressant drug mycophenolate mofetil (MMF) by using (3-aminopropyl) triethoxysilane (APTES)-functionalized Nb_2_CT_*x*_ MXene nanosheets (Nb_2_CT_*x*_-APES) modified carbon cloth is reported^22^ which could detect MMF over the range of 10–100 µM. A promising electrochemical sensor for L-cysteine was developed by depositing platinum nanoparticles on Nb_2_CT_*x*_ MXene^23^.

Selenium (Se) nanoparticles are known for their excellent catalytic activity, good biocompatibility, and ability to facilitate electron transfer, all of which contribute to the superior sensitivity and selectivity of the composite for glucose detection. Nb_2_CT_*x*_ MXene has been successfully combined with Se to create composite materials with enhanced electrocatalytic properties^24,25^. The synergistic combination of MXene’s high conductivity and large surface area with selenium’s catalytic performance results in a highly efficient sensing platform.

This work reports the synthesis of Nb_2_CT_*x*_ MXene incorporated with Se nanoparticles followed by its application towards glucose detection in an alkaline medium. Se nanoparticles were incorporated into the MXene layers through ultrasonication. The sensor was fabricated by incorporating the nanocomposite on a gold electrode. The developed sensor could detect a wide range of glucose concentrations in 0.1 M NaOH.

## Experimental

### Materials and instrumentation

MAX phase Nb_2_AlC, sodium selenite (99%), dopamine hydrochloride, ascorbic acid, and glucose (99%) were purchased from Sigma Aldrich. Uric acid (99%) was purchased from Loba Chemie. 48% HF, potassium chloride (KCl, 99%), sodium hydroxide (NaOH, 97%), and potassium ferricyanide (K_3_[Fe(CN)_6_], (98%) were purchased from Finar. De-ionized water (15 MΩ cm^−1^) from Elix-10 (Millipore, Germany) was used to prepare solutions for all experiments.

The surface morphology of the nanocomposite was studied using Field Emission Scanning Electron Microscopy (FESEM) (GEMINI 300, Carl Zeiss, Germany) provides resolution of the images as low as 2 nm at 15 kV, and elemental characterization was performed using X-ray diffractometer (ULTIMA IV, Rigaku, Japan) (Cu K_α_). To analyze the functional groups, present in the nanocomposite Raman microscopy (LABRAM HR evolution, HORIBA Jobin–Yvon, France) with laser wavelength of 532 nm and X-Ray photoelectron microscopy (XPS) (Thermofisher, K-alpha, USA) was used. All the electrochemical studies were conducted using CHI 6088D (CH Instruments, USA) using a three-electrode arrangement.

### Synthesis of Nb_2_CT_***x***_ MXenes

Synthesis of Nb_2_CT_*x*_ nanosheets was carried out as reported^26^. Nb_2_AlC MAX phase (1 g) was added gradually to 48% HF (20 mL) and stirred for 96 h at room temperature in a polypropylene container. The etched Nb_2_CT_*x*_ (e-Nb_2_CT_*x*_) was washed several times with de-ionized water (DI) water until the pH became 6 and was dried at 50 °C. Delamination of e-Nb_2_CT_*x*_ was achieved by ultrasonication for 1 h. The delaminated Nb_2_CT_*x*_ (d-Nb_2_CT_*x*_) was dried at 50 °C and stored in an air-tight container in the refrigerator until further use to avoid oxidation.

### Synthesis of selenium nanoparticles (SeNP)

SeNP were synthesized by reducing sodium selenite (Na_2_SeO_3_) with ascorbic acid as reported in other works^27,28^. 2.5 mM Na_2_SeO_3_ was prepared and stirred at 90 °C for 1 h. To this solution, 2 M L-ascorbic acid was added dropwise and stirred for 10 min. The formed product was collected by centrifugation at 8000 rpm, washed multiple times with water, and dried overnight at 60 °C.

### Synthesis of Nb_2_CT_***x***_@Se nanocomposites and fabrication of the sensor electrode

**Fig. 1:**
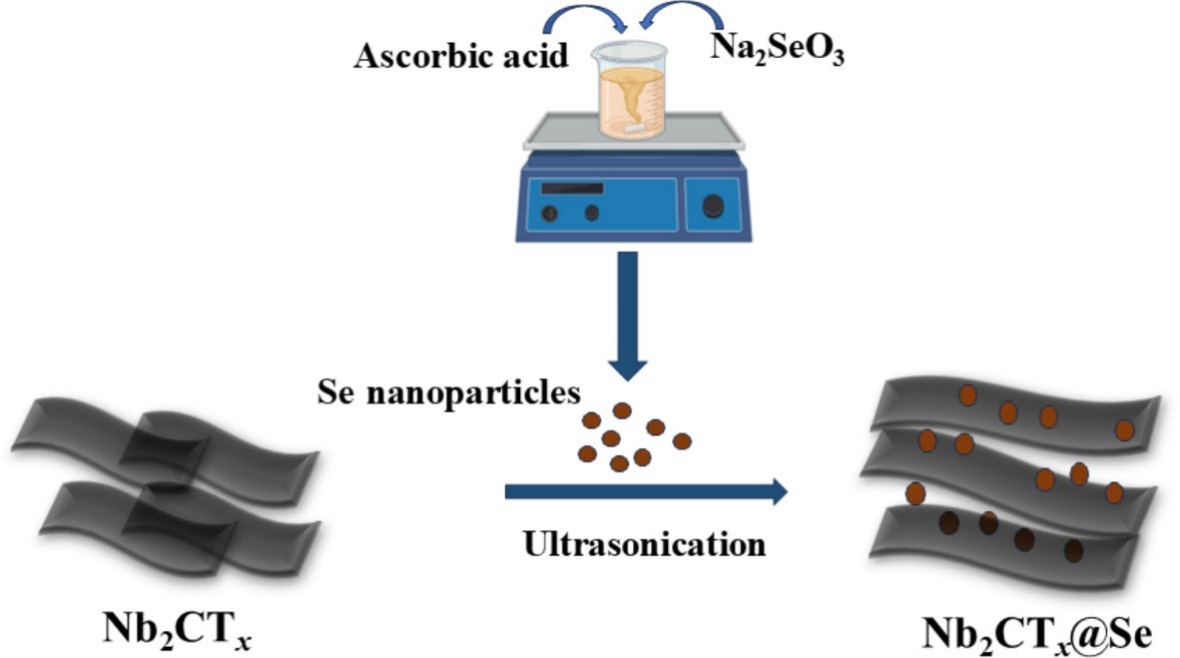
Synthesis of Nb_2_CT_x_@Se.

20 mg of the SeNP and 20 mg of d-Nb_2_CT_*x*_ were mixed with 40 mL of ethanol and ultrasonicated for 1 h. The product was rinsed with water and labelled as Nb_2_CT_*x*_@50Se according to the amount of SeNP added. The obtained precipitate was then dried at 50 °C overnight. Similarly, Nb_2_CT_*x*_@Se with 25% and 75% selenium nanoparticles were synthesized, indicated as Nb_2_CT_*x*_@25Se and Nb_2_CT_*x*_@75Se, respectively (Fig. 1).

Gold (Au) disc electrodes were cleaned and polished using 0.05-micron alumina powder followed by ultrasonication in DI water. 1 mg of each Nb_2_CT_*x*_@Se nanocomposite was dispersed in 1 mL DI water, and sonicated for 1 h (1 mg/mL). 2 µL of this suspension was then drop-casted onto the Au electrode and dried at 50 °C for 10 min^29^. The electrode was thereby named as Nb_2_CT_*x*_@Se/Au.

### Electrochemical characterization

The developed sensor was characterized electrochemically in 0.1 M NaOH with Nb_2_CT_*x*_@Se/Au, Ag/AgCl, platinum wire as the working electrode, reference electrode, and the counter electrode, respectively. To study the electrochemical behaviour of glucose on the modified electrode, initially linear sweep voltammetry (LSV) was performed within a potential window of − 0.3 to 0.25 V at a scan rate of 0.05 V s^−1^ using the three-electrode assembly. The response was further evaluated using sensitive technique like differential pulse voltammetry (DPV) which was carried out between − 0.15 and 0.2 V. From the voltammetric analysis, the amperometric potential was chosen as 0.16 V. The sensor performance was evaluated amperometrically in 0.1 M NaOH with various glucose concentrations and the current response obtained at the 9^th^ second was utilized for quantification.

## Results and discussion

### Elemental and morphological characterisation of Nb_2_CT_x_@50Se

**Fig. 2:**
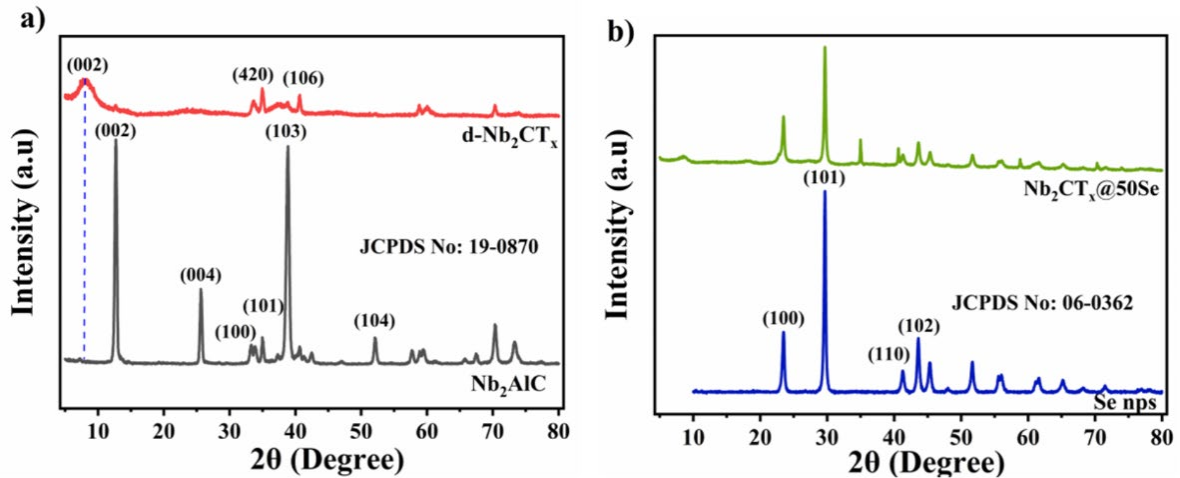
X-ray diffractogram for (**a**) Nb_2_AlC and d-Nb_2_CT_x_, (**b**) SeNP and Nb_2_CT_x_@50Se.

To understand the formation of d-Nb_2_CT_*x*_ from Nb_2_AlC, the XRD pattern of the MAX phase before and after delamination was recorded (Fig. 2a). The formation of the delaminated MXene sheets from the MAX phase was confirmed by the characteristic peak shift of the (002) plane from 12.6° to 7.8°. The broadening and shifting of the (002) peak to lower angles is due increase in interlayer distance or c lattice parameter and the diminishing of the MAX peaks confirms the successful formation and delamination of the MXenes^30^. To confirm the incorporation of SeNP onto the MXene layers, the XRD pattern of SeNP and Nb_2_CT_*x*_@50Se was recorded (Fig. 2b). The distinctive peaks at 23.5°, 29.7°, 41.4°, 43.6°, 45.4°, 51.7°, 55.9°, and 61.5° correspond to (100), (101), (110), (102), (111), (201), (112), and (202) planes of Se^28^. The diffraction peaks corresponding to that of SeNP were found in that of the composite, thereby, confirming the formation of Nb_2_CT_*x*_@50Se.

**Fig. 3:**
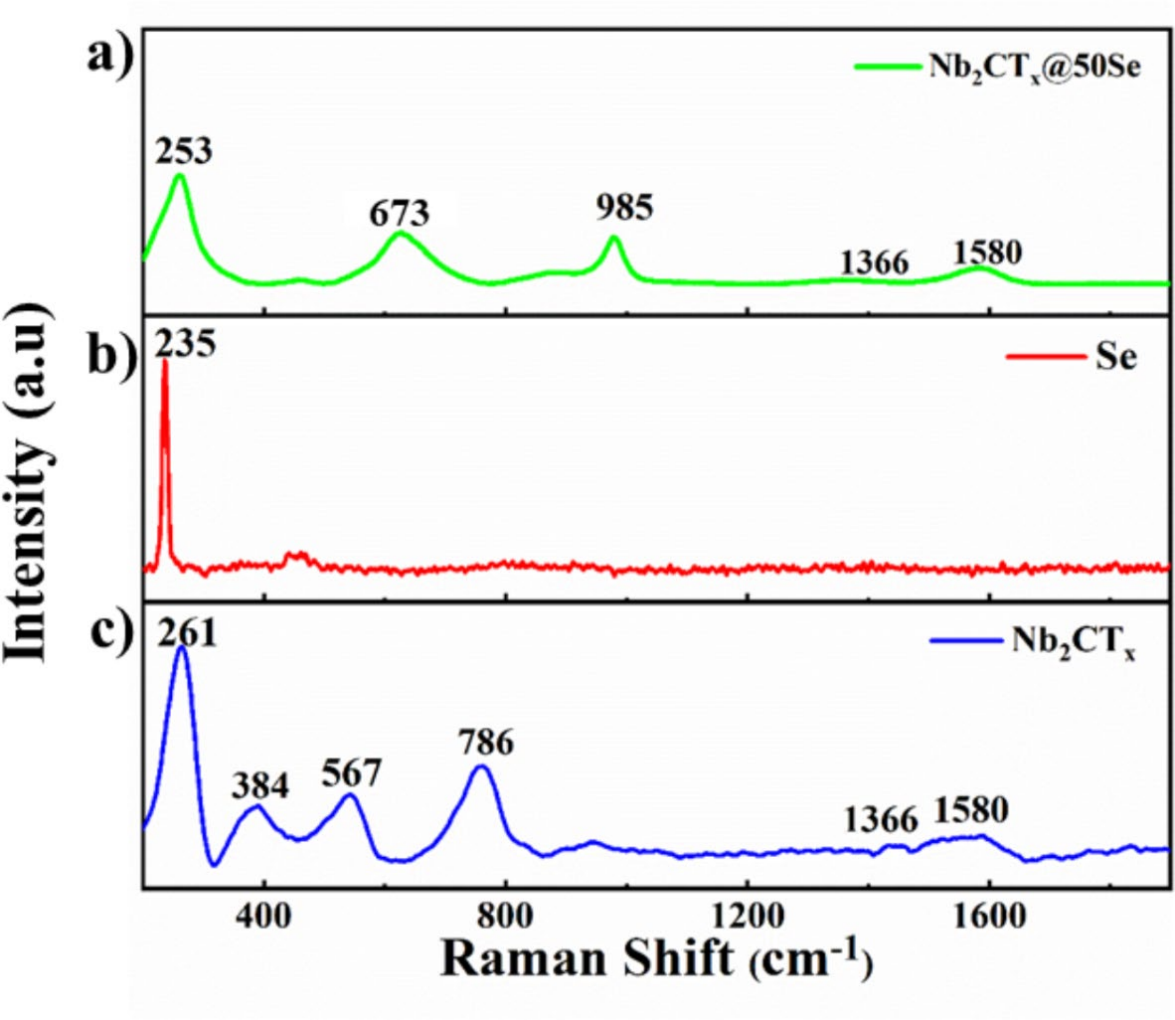
Raman spectra of d-Nb_2_CT_x_, SeNP, and Nb_2_CT_x_@50Se.

Raman spectra of Nb_2_CT_*x*_, SeNP, and Nb_2_CT_*x*_@50Se were recorded between 150 and 2000 cm^−1^ (Fig. 3). The characteristic peak at 261 cm^−1^ in the spectrum of Nb_2_CT_*x*_ corresponds to the A_1g_ mode of symmetrical out-of-plane vibrations of the Nb-C bond (Fig. 3c). Peaks at 1366 cm^−1^ and 1580 cm^−1^ correspond to the D band (due to the disorderliness of carbon species of Nb_2_CT_*x*_) and G band (due to the vibrations of sp^2^ hybridized carbon in Nb_2_CT_*x*_), respectively which was observed in both Nb_2_CT_*x*_ and Nb_2_CT_*x*_@50Se (Fig. 3a). The D and G bands were distinctively observed in Nb_2_CT_*x*_@50Se which is an indication of the surface modification of Nb_2_CT_*x*_. In Fig. 3b the Se–Se bond vibration was observed at 235 cm^−1^^31^. The peak at 673 cm^−1^ of the composite corresponds to Se–C vibration^32^, which indicates the formation of Nb_2_CT_*x*_@50Se. Additional peaks observed in the case of Nb_2_CT_*x*_ at 384 cm^−1^, 567 cm^−1^, and 786 cm^−1^ correspond to characteristic peaks for the Si substrate used for the analysis^33^.

**Fig. 4:**
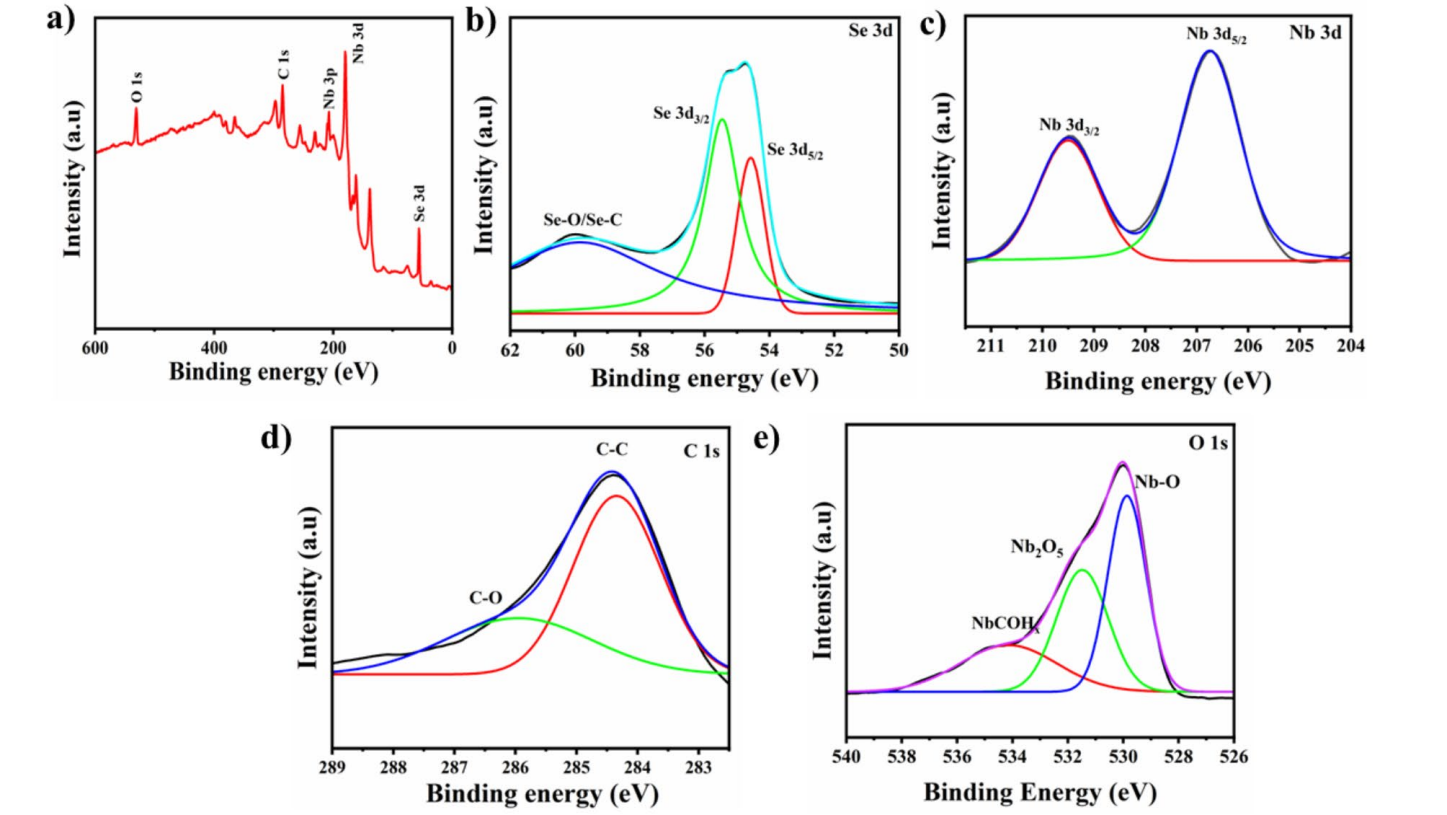
(**a**) XPS spectra of Nb_2_CT_x_@50Se, high resolution spectra of (**b**) Se 3d, (**c**) Nb 3d, and (**d**) O* 1 s.*

XPS analysis of the composite was performed to study the elemental characteristics. The survey spectrum of Nb_2_CT_*x*_@50Se shows the presence of Se, C, O, and Nb confirming the formation of the composite (Fig. 4a)^34^. The high-resolution spectrum of Se 3d (Fig. 4b) confirms the presence of the Se–C bond at 60 eV and the peaks located at 54.7 corresponds to Se 3d_5/2_ and that at 55.6 eV is attributed to Se 3d_3/2_^35^. Peaks at 207 eV and 210 eV in high resolution spectra of Nb 3d (Fig. 4c) correspond to Nb 3d_3/2_ and Nb 3d_5/2,_ respectively^36^. The result attributes to the electron distribution of Nb 3d that changes due to the incorporation of SeNP, which also affects the bonding properties of Nb. C 1s spectra (Fig. 4d) show peaks at 284.4 eV and 286 eV which corresponds to C–C and C–O bonds^37^. High resolution O 1s (Fig. 4e) spectra has a peak at 530.1, 532.3, and 534 eV corresponding to Nb_2_O_5_, NbCO and Nb_2_C(OH)_x_, respectively, which indicates the existence of –OH and –O groups at the surface termination of Nb_2_CT_*x*_^38^.

**Fig. 5:**
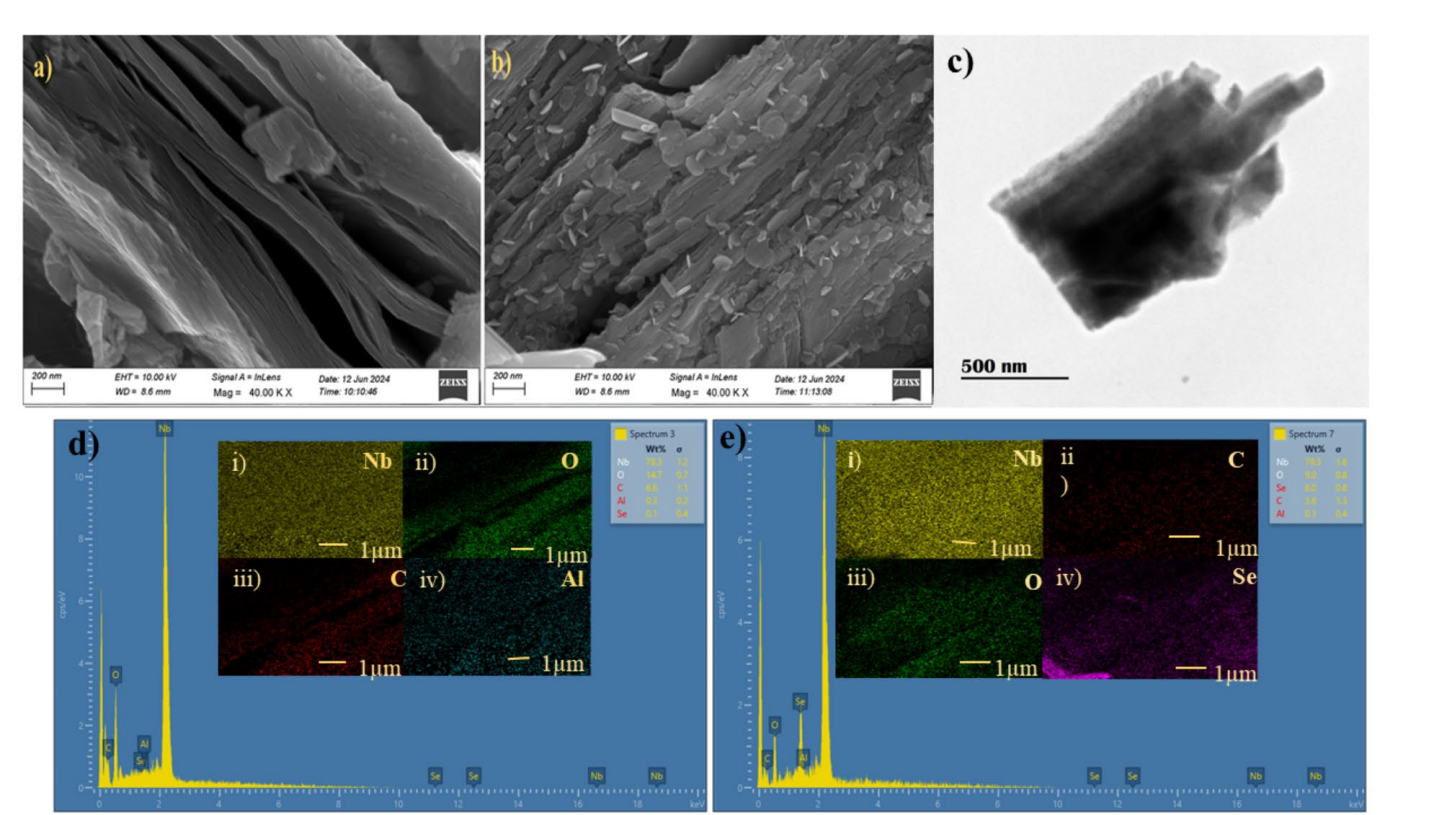
FESEM images for (**a**) Nb_2_CT_x_ and (**b**) Nb_2_CT_x_@50Se. (**c**) TEM image for Nb_2_CT_x_@50Se. EDX spectrum and elemental mapping for (**d**) Nb_2_CT_x_ and e) Nb_2_CT_x_@50Se.

The surface morphology of synthesized Nb_2_CT_*x*_ and Nb_2_CT_*x*_@50Se were examined in FESEM. The FESEM image of Nb_2_CT_*x*_ (Fig. 5a) which shows layered and sheet-like structures that indicate the formation of MXene from the Nb_2_AlC MAX phase. These layered sheets appear to be orderly stacked with a small number of impurities such as Al, and Si as indicated in the EDX (Fig. 5d)^39^. The presence of impurities was indicated by the Raman spectrum also. The FESEM image of Nb_2_CT_*x*_@50Se (Fig. 5b) indicates the layered structure of MXene with SeNP on it. The incorporation of SeNP onto the MXene layers was further confirmed using EDX (Fig. 5e), where the proportion of Se was found to be 8%. The surface of the synthesized Nb_2_CT_*x*_@50Se was further evaluated using TEM. The Fig. 5c indicates the sheet like structure of the MXene and the incorporation of SeNPs onto the Nb_2_CT_*x*_ MXene layers.

### Electrochemical characterization of Nb_2_CT_x_@Se/Au towards glucose detection

The Au electrode and that modified using Nb_2_CT_*x*_@Se were tested using various voltammetric techniques towards glucose detection. Fig. S1a indicates the current response obtained towards glucose in 0.1 M NaOH obtained on Au, Nb_2_CT_*x*_/Au and Nb_2_CT_*x*_@50Se/Au. A strong anodic peak was observed only in the case of Nb_2_CT_*x*_@50Se/Au which indicates that the incorporation of SeNP is responsible for the oxidation response of glucose. The effect of SeNP loading on the Nb_2_CT_*x*_ was studied from the response obtained on Au electrode modified using the composite containing various amounts of SeNP towards glucose in 0.1 M NaOH (Fig. S1b). The results indicate that the maximum response towards glucose was obtained when the amount of SeNP loaded on Nb_2_CT_*x*_ is 50%, which was chosen as optimum.

The electrolyte concentration is one of the crucial factors in the performance of a sensor. To optimize the concentration of the electrolyte solution, the current response on Nb_2_CT_*x*_@50Se/Au towards glucose was recorded in various concentrations of NaOH such as 0.05 M, 0.1 M, and 0.15 M (Fig. S1c). The results indicate that the maximum response was obtained in 0.1 M NaOH and was chosen as the optimum electrolyte concentration. The amount of catalytic material present on the electrode surface is directly associated to the current response. The effect of the amount of the catalytic material loaded on the electrode surface towards glucose response was studied and the results are shown in Fig. S1d. The results indicate that 2 µL of the catalytic material when used to modify the electrode surface showed the highest current response. When a large amount of catalytic material is incorporated on the electrode surface a decrease in current response is observed, possibly due to decrease of the effective surface area.

**Fig. 6:**
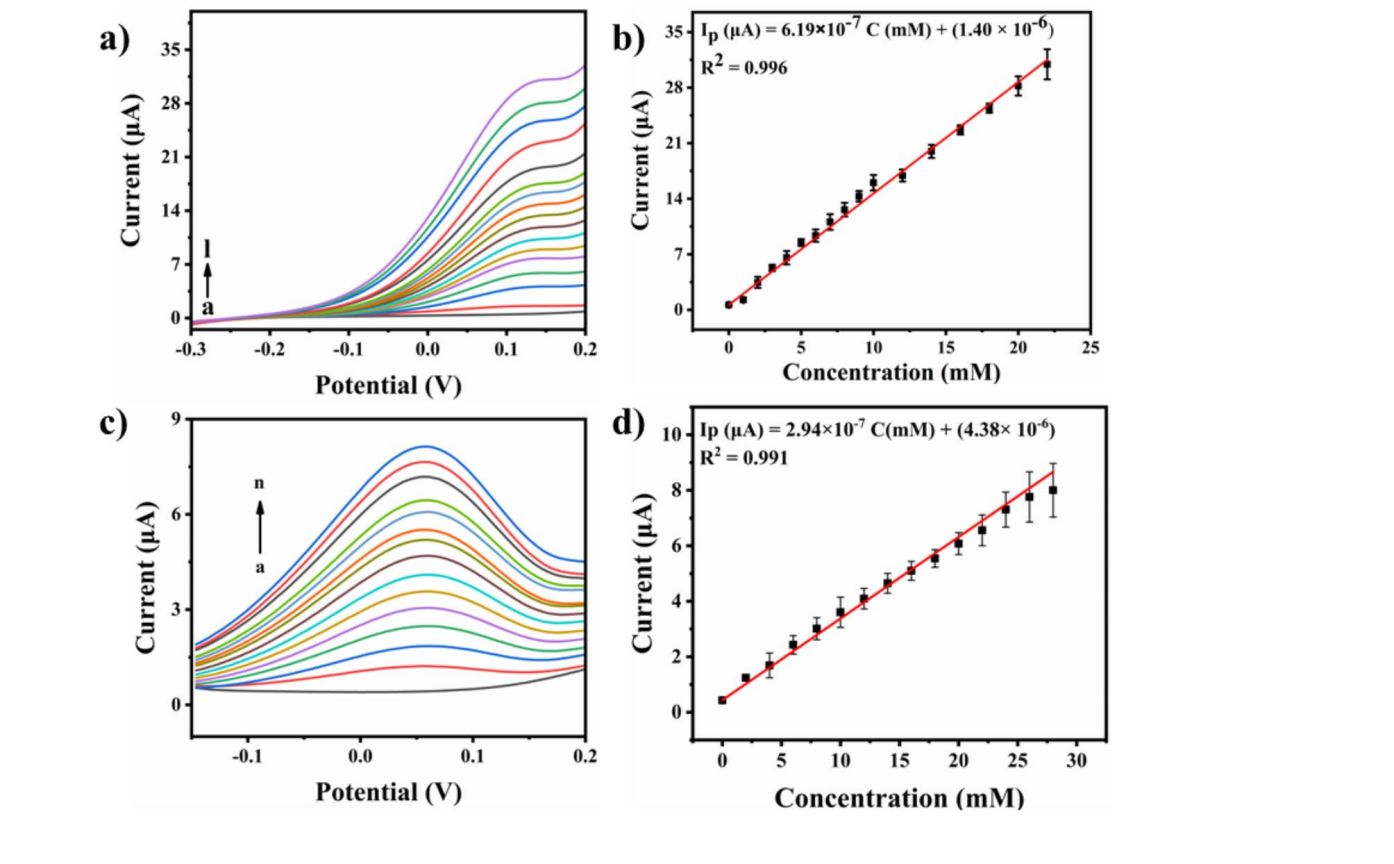
LSV of Nb_2_CT_x_@50Se/Au in 0.1 M NaOH (**a**) with various concentrations of glucose from 2 to 22 mM (a-l). (**b**) Linear plot between concentrations of glucose and peak current. (**c**) DPV of Nb_2_CT_x_@50Se/Au with different concentrations of glucose 2–28 mM. (**d**) Linear plot between concentrations of glucose and peak current 0.1 M NaOH.

Figure 6a shows the LSVs on Nb_2_CT_*x*_@50Se/Au in 0.1 M NaOH containing different glucose concentrations at a scan rate of 0.05 V s^−1^. An anodic peak at 0.15 V was observed that corresponds to glucose oxidation. The current response was found to increase with glucose concentration from 2 to 22 mM. The peak current was found to increase linearly with linear regression equation I_p_ (μA) = (6.19 × 10^–7^) C (mM) + (1.40 × 10^–6^) with R^2^ = 0.996 (Fig. 6b).

To evaluate the glucose oxidation a more sensitive electroanalytical technique such as DPV on Nb_2_CT_*x*_@50Se/Au in 0.1 M NaOH between − 0.15 and 0.2 V was recorded. A well-defined anodic peak was observed at around 0.05 V corresponding to glucose oxidation. The results indicate that the current increase with concentration up to 28 mM (Fig. 6c). The peak current was found to increase linearly with concentration with a linear equation of Ip (μA) = 2.94 × 10^–7^ C(mM) + (4.38 × 10^–6^) and R^2^ = 0.991 (Fig. 6d).

**Fig. 7:**
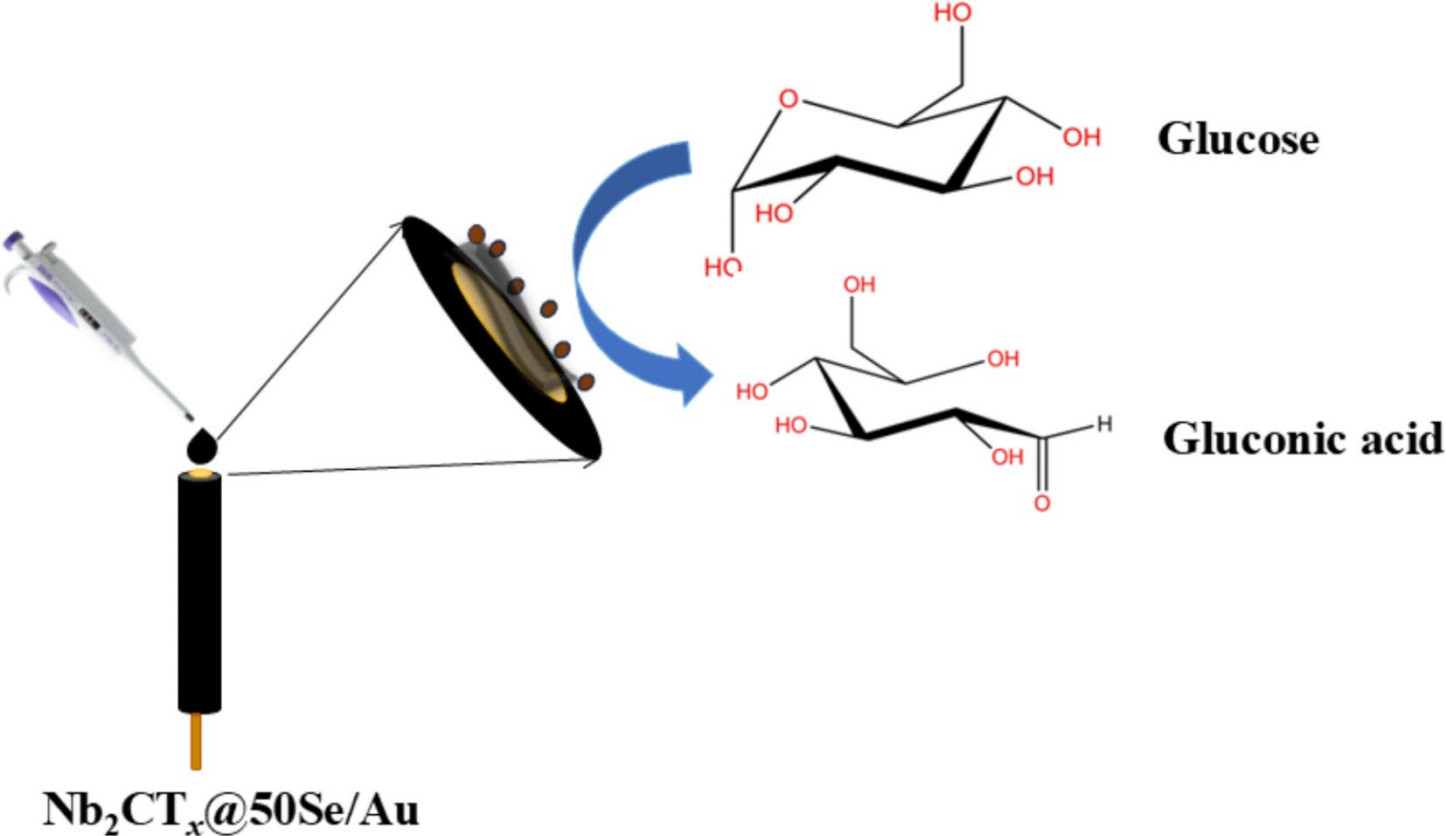
Glucose oxidation of the electrocatalytic material.

The electrocatalytic material provide active sites for molecule adsorption and electron transfer which is the reason for the current response towards glucose oxidation (Fig. 7). The electrocatalytic activity of SeNP is enhanced by the incorporation of MXene. The synergy between Nb_2_CT_*x*_ and SeNP paves way for the glucose oxidation at a lower overpotential when compared with other reports on glucose oxidation in alkaline medium^40^.

During the reaction, Se^0^ was reduced to Se^2−^ (Eq. 1). The oxidation peak at 0.15 V corresponds to the oxidation of glucose to gluconic acid. Glucose adsorbs to the surface of SeNP, and oxidizes, aided by the alkaline environment and the catalytic properties of the nanocomposite. Electrons produced in this process were transferred to the electrode, generating a detectable current. The resulting gluconate then detaches from the SeNP and hydrolyzes to form gluconic acid^41,42^.

1 $${Se}^{0} +{2e}^{-} \to {Se}^{2-}$$

To study the mechanism of the glucose oxidation on Nb_2_CT_*x*_@50Se/Au, LSVs at various scan rates were performed in 0.1 M NaOH containing 6 mM glucose (Fig. S2a). The current response increases with the scan rate. The peak current response was found to increase linearly with the square root of scan rate obeying the equation I_p_ (μA) = (2.86 × 10^–6^) √υ (Vs^−1^)^1/2^ + (− 4.82 × 10^–6^) and R^2^ = 0.993 (Fig. S2b). The results indicate that the glucose oxidation on Nb_2_CT_*x*_@50Se/Au is a diffusion-controlled reaction^43^. 

### Electrochemical quantification of glucose

**Fig. 8:**
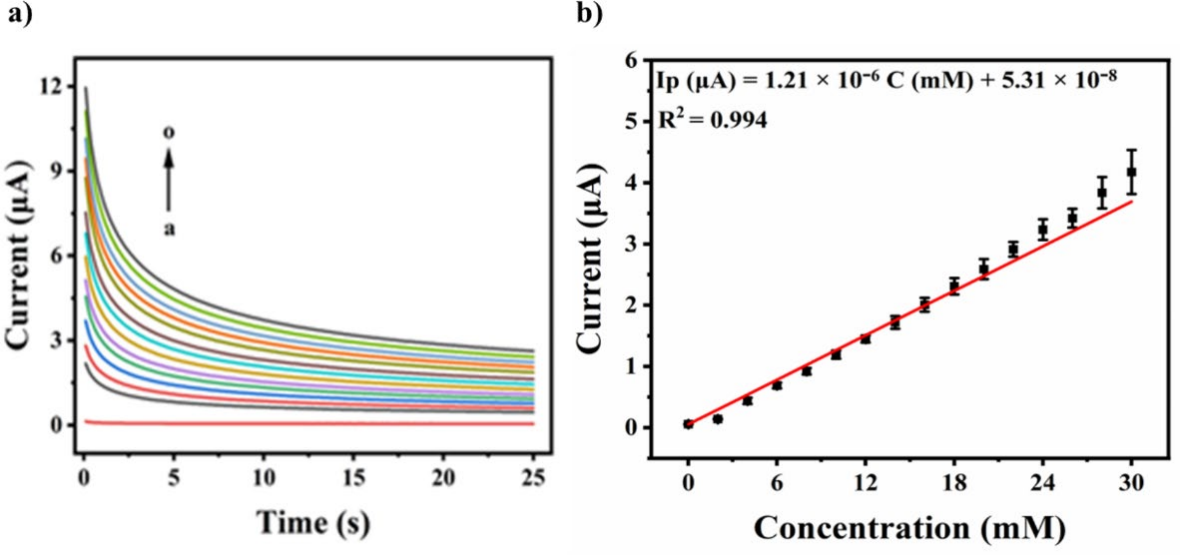
(**a**) Amperometric response at 0.16 V on Nb_2_CT_x_@50Se/Au in 0.1 M NaOH containing glucose from 2 to 30 mM, (**b**) Calibration plot between concentration and current.

The amperometric response on Nb_2_CT_*x*_@50Se/Au in 0.1 M NaOH containing different glucose concentrations was performed at 0.16 V (Fig. 8a). The results show that the current response increases up to 30 mM. The current response corresponding to the 9th second was plotted against glucose concentrations and was found to increase linearly with the regression equation I_p_ (µM) = (1.21 × 10^–6^) C (mM) + (5.31 × 10^–8^) and R^2^ value 0.994 (Fig. 8b). Reported works on glucose detection in alkaline medium indicate the oxidation potential around 0.5 V. However, with the Nb_2_CT_*x*_@50Se electrocatalyst, the oxidation potential of glucose was brought down to 0.16 V in alkaline medium, which is the major highlight of this work. The sensitivity was calculated from the slope of the linear plot and was found to be 4.15 µA mM^−1^ cm^−2^. The limit of detection (LOD) was statistically determined as 1.1 mM.

### Selectivity and reproducibility

 The current response of 12 mM glucose on Nb_2_CT_*x*_@50Se/Au in 0.1 M NaOH in the presence of potentially interfering molecules like dopamine (100 µM), glycine (0.25 mM), uric acid (10 µM), creatinine (200 µM) and ascorbic acid (100 µM) were recorded. Figure S3a depicts the percentage current response of the interfering molecules relative to that of glucose. The results show that all the molecules except creatinine show a current response below 10%, which is highly promising as the developed sensor is selective towards glucose.

To evaluate the reproducibility of the developed sensor, the current response from ten different electrodes were recorded and the result is depicted in Fig. S3b. There was no significant change in the current response (only 2.8% of standard deviation) observed for ten different electrodes, which showcases the reproducibility of the sensor.

## Conclusion

The work presents the synthesis of Nb-based MXene-Se nanocomposite for selective electrochemical detection of glucose in alkaline medium. The synergistic effect between Nb_2_CT_*x*_ and SeNP was utilized for the enhanced response towards glucose. The oxidation was observed at 0.16 V in 0.1 M NaOH which is relatively lower overpotential compared to the previous works. The developed sensor exhibited a sensitivity of 4.15 µA mM^−1^ cm^−2^ with a linear detection range of 2–30 mM with a detection limit of 1.1 mM. The evaluation of the sensor performance in biofluid samples like blood serum will provide an insight towards its real time application. In order to imply the sensing mechanism to point of care testing, the catalytic material can be incorporated onto disposable printed electrodes which is another point of advancement.

## Electronic supplementary material

Below is the link to the electronic supplementary material.

Supplementary Material 1

## Data Availability

The data that support the findings of this study are available on request from the corresponding author, T G Satheesh Babu, upon reasonable request.

## References

[1] Cagnani, G. R. et al. From research to market: correlation between publications, patent filings, and investments in development and production of technological innovations in biosensors. *Anal. Bioanal. Chem.* **415**, 3645–3653. 10.1007/s00216-022-04444-2 (2023). 36477496 10.1007/s00216-022-04444-2 PMC9734492

[2] Glucose Biosensors Market Size And Share Report, 2030, https://www.grandviewresearch.com/industry-analysis/glucose-biosensors-market (2024).

[3] Prakash, M. D., Nihal, S. L., Ahmadsaidulu, S., Swain, R. & Panigrahy, A. K. Design and modelling of highly sensitive glucose biosensor for lab-on-chip applications. *Silicon* **14**, 8621–8627. 10.1007/s12633-021-01543-0 (2022).

[4] Saklani, S. et al. Nanomaterials-integrated electrochemical biosensors as pioneering solutions for zoonotic disease diagnosis. *J. Electrochem. Soc.* **171**, 087502. 10.1149/1945-7111/ad65bb (2024).

[5] Hou, S., Zhang, A. & Su, M. Nanomaterials for biosensing applications. *Nanomaterials* **6**, 58. 10.3390/nano6040058 (2016). 28335185 10.3390/nano6040058 PMC5302573

[6] Ahmed, A. M. et al. Advances in CuO nanostructure-based glucose biosensors: From enzymes to direct oxidation. *NANO*10.1142/S1793292024300135 (2024).

[7] Dhara, K., Stanley, J., Ramachandran, T., Nair, B. G. & Satheesh Babu, T. G. Pt-CuO nanoparticles decorated reduced graphene oxide for the fabrication of highly sensitive non-enzymatic disposable glucose sensor. *Sens. Actuators B Chem.* **195**, 197–205. 10.1016/j.snb.2014.01.044 (2014).

[8] Chandhana, J. P. et al. Zirconium copper oxide microflowers based non-enzymatic screen-printed electrochemical sensor for the detection of glucose in saliva, urine, and blood serum. *Microchim. Acta*10.1007/s00604-023-05965-y (2023). 10.1007/s00604-023-05965-y 37700117

[9] Meera, R. et al. Non-enzymatic electrochemical detection of urine creatinine using cobalt-gold bimetallic nanoparticles. *J. Electrochem. Soc.* **171**(6), 067504. 10.1149/1945-7111/ad4e71 (2024).

[10] Neena, P. K. et al. Alkaline hematin-based disposable electrochemical sensor for haemoglobin. *J. Electrochem. Soc.* **170**, 087515. 10.1149/1945-7111/acf075 (2023).

[11] Kumar, A., Suneesh, P. V., Nair, B. K. G. & Satheesh Babu, T. G. Complete fabrication of a nonenzymatic glucose sensor with a wide linear range for the direct testing of blood samples. *Electrochim. Acta* **395**, 139145. 10.1016/j.electacta.2021.139145 (2021).

[12] Chaudhary, V., Talreja, R. K., Sonu, S., Rustagi, R. & Walvekar, A. G. High-performance H2 sensor based on Polyaniline-WO3 nanocomposite for portable batteries and breathomics-diagnosis of irritable bowel syndrome. *Int. J. Hydrog. Energy* **52**, 1156–1163. 10.1016/j.ijhydene.2023.08.151 (2024).

[13] Luo, J., Jiang, S., Zhang, H., Jiang, J. & Liu, X. A novel non-enzymatic glucose sensor based on Cu nanoparticle modified graphene sheets electrode. *Anal. Chim. Acta* **709**, 47–53. 10.1016/j.aca.2011.10.025 (2012). 22122930 10.1016/j.aca.2011.10.025

[14] Chaudhary, V. et al. Borophene-based nanomaterials: Promising candidates for next-generation gas/vapor chemiresistors. *J. Mater. Sci. Technol.* **218**, 236–262. 10.1016/j.jmst.2024.08.038 (2025).

[15] Lu, M. et al. Core-shell MOF@MOF composites for sensitive nonenzymatic glucose sensing in human serum. *Anal. Chim. Acta* **1110**, 35–43. 10.1016/j.aca.2020.02.023 (2020). 32278398 10.1016/j.aca.2020.02.023

[16] Khosla, A. et al. Emergence of MXene and MXene–polymer hybrid membranes as future-environmental remediation strategies. *Adv. Sci.* **9**, 36. 10.1002/advs.202203527 (2022). 10.1002/advs.202203527 PMC9798995 36316226

[17] VahidMohammadi, A., Rosen, J. & Gogotsi, Y. The world of two-dimensional carbides and nitrides (MXenes). *Science* **372**, 1581. 10.1126/science.abf1581 (2021). 10.1126/science.abf1581 34112665

[18] Naguib, M., Barsoum, M. W. & Gogotsi, Y. Ten years of progress in the synthesis and development of MXenes. *Adv. Mater.* **33**, 2103393. 10.1002/adma.202103393 (2021). 10.1002/adma.202103393 34396592

[19] Gogotsi, Y. & Huang, Q. MXenes: Two-dimensional building blocks for future materials and devices. *ACS Nano* **15**, 5775–5780. 10.1021/acsnano.1c03161 (2021). 33906288 10.1021/acsnano.1c03161

[20] Arjun, A. M. et al. An overview on surface modification of niobium MXenes for diagnostic and prognostic applications. *FlatChem* **41**, 100538. 10.1016/j.flatc.2023.100538 (2023).

[21] Ankitha, M., Shabana, N., Mohan Arjun, A., Muhsin, P. & Abdul Rasheed, P. Ultrasensitive electrochemical detection of dopamine from human serum samples by Nb2CTx-MoS2 hetero structures. *Microchem. J.* **187**, 108424. 10.1016/j.microc.2023.108424 (2023).

[22] Vaishag, P. V., Ankitha, M. & Rasheed, P. A. Carbon cloth yarn modified with amino-functionalized Nb2CTx nanosheets for the electrochemical detection of mycophenolate mofetil. *ACS Appl. Nano Mater.* **6**, 23324–23331. 10.1021/acsanm.3c04679 (2023).

[23] Shabana, N., Ankitha, M., Arjun, A. M. & Rasheed, P. A. In-situ decoration of platinum nanoparticles on Nb2CTx MXene: An electrochemical sensor for l-cysteine and an efficient catalyst for oxygen evolution reaction. *ECS J. Solid State Sci. Technol.* **11**, 127002. 10.1149/2162-8777/aca793 (2022).

[24] Saji, V. S. & Lee, C.-W. Selenium electrochemistry. *RSC Adv.* **3**, 10058–10077. 10.1039/C3RA40678D (2013).

[25] Wei, Y. et al. Selenium-based catalysts for efficient electrocatalysis. *Adv. Funct. Mater.*10.1002/adfm.202404787 (2024).

[26] Mohan Arjun, A., Shabana, N., Ankitha, M. & Abdul Rasheed, P. Electrochemical deposition of Prussian blue on Nb2CTx MXene modified carbon cloth for the non-enzymatic electrochemical detection of hydrogen peroxide. *Microchem. J.* **185**, 108301. 10.1016/j.microc.2022.108301 (2023).

[27] Vahdati, M. & Tohidi Moghadam, T. Synthesis and characterization of selenium nanoparticles-lysozyme nanohybrid system with synergistic antibacterial properties. *Sci. Rep.* **10**, 510. 10.1038/s41598-019-57333-7 (2020). 31949299 10.1038/s41598-019-57333-7 PMC6965607

[28] Shar, A. H. et al. Facile synthesis and characterization of selenium nanoparticles by the hydrothermal approach. *Dig. J. Nanomater. Biostruct.* **14**, 867–872 (2019).

[29] Divya, K. P., Keerthana, S., Viswanathan, C. & Ponpandian, N. MXene supported biomimetic bilayer lipid membrane biosensor for zeptomole detection of BRCA1 gene. *Microchim. Acta* **190**, 116. 10.1007/s00604-023-05694-2 (2023). 10.1007/s00604-023-05694-2 36877256

[30] Rasheed, P. A., Pandey, R. P., Banat, F. & Hasan, S. W. Recent advances in niobium MXenes: Synthesis, properties, and emerging applications. *Matter* **5**, 546–572. 10.1016/j.matt.2021.12.021 (2022).

[31] Wang, T., Yang, L., Zhang, B. & Liu, J. Extracellular biosynthesis and transformation of selenium nanoparticles and application in H_2_O_2_ biosensor. *Colloids Surf. B Biointerfaces* **80**, 94–102. 10.1016/j.colsurfb.2010.05.041 (2010). 20566271 10.1016/j.colsurfb.2010.05.041

[32] Li, Y. et al. Stable carbon-selenium bonds for enhanced performance in tremella-like 2D chalcogenide battery anode. *Adv. Energy Mater.* **8**, 1800927. 10.1002/aenm.201800927 (2018).

[33] Lin, H., Gao, S., Dai, C., Chen, Y. & Shi, J. A two-dimensional biodegradable niobium carbide (MXene) for photothermal tumor eradication in NIR-I and NIR-II biowindows. *J. Am. Chem. Soc.* **139**, 16235–16247. 10.1021/jacs.7b07818 (2017). 29063760 10.1021/jacs.7b07818

[34] Shen, B. et al. Rosette-like (Ni, Co)Se2@Nb2CTx MXene heterostructure with abundant Se vacancies for high-performance flexible supercapacitor electrodes. *Chem. Eng. J.* **484**, 149440. 10.1016/j.cej.2024.149440 (2024).

[35] Li, J. et al. Wang, synergy of MXene with Se infiltrated porous N-doped carbon nanofibers as janus electrodes for high-performance sodium/lithium–selenium batteries. *Adv. Energy Mater.* **12**, 2200894. 10.1002/aenm.202200894 (2022).

[36] Su, T. et al. One-step synthesis of Nb_2_O_5_/C/Nb_2_C (MXene) composites and their use as photocatalysts for hydrogen evolution. *ChemSusChem* **11**, 688–699. 10.1002/cssc.201702317 (2018). 29281767 10.1002/cssc.201702317

[37] Zeng, G., Zhou, J. & Ren, S. Two-dimensional Nb2CTx nanosheets decorated LiFePO_4_/C as cathode material for lithium-ion batteries. *J. Mater. Sci.* **58**, 5413–5426. 10.1007/s10853-023-08361-2 (2023).

[38] Xiao, J. et al. A safe etching route to synthesize highly crystalline Nb2CTx MXene for high performance asymmetric supercapacitor applications. *Electrochim. Acta* **337**, 135803. 10.1016/j.electacta.2020.135803 (2020).

[39] Gul, S. et al. Un-doped and Er-adsorbed layered Nb2C MXene for efficient hydrazine sensing application. *Surf. Interfaces* **24**, 101074. 10.1016/j.surfin.2021.101074 (2021).

[40] Chu, D. et al. Prism-like bimetallic (Ni–Co) alkaline carboxylate-based non-enzymatic sensor capable of exceptionally high catalytic activity towards glucose. *Dalton Trans.* **51**, 15354–15360. 10.1039/D2DT02424A (2022). 36148531 10.1039/d2dt02424a

[41] Hao, X., Jia, J., Chang, Y., Jia, M. & Wen, Z. Monodisperse copper selenide nanoparticles for ultrasensitive and selective non-enzymatic glucose biosensor. *Electrochim. Acta* **327**, 135020. 10.1016/j.electacta.2019.135020 (2019).

[42] Bisht, N., Phalswal, P. & Khanna, P. K. Selenium nanoparticles: A review on synthesis and biomedical applications. *Mater. Adv.* **3**, 1415–1431. 10.1039/D1MA00639H (2022).

[43] Ashok, A., Kumar, A. & Tarlochan, F. Highly efficient nonenzymatic glucose sensors based on CuO nanoparticles. *Appl. Surf. Sci.* **481**, 712–722. 10.1016/j.apsusc.2019.03.157 (2019).

