# Supplementary material for: Selenium nanoparticles modified niobium MXene for non-enzymatic detection of glucose
Source: Sci Rep. 2025 Jan 11;15:1749. doi: 10.1038/s41598-025-85748-y (PMC11724989; doi:10.1038/s41598-025-85748-y)
Supplement: Supplementary file 1 — Supplementary Material 1 [file 41598_2025_85748_MOESM1_ESM.docx]

**Selenium nanoparticles modified Niobium MXene for non-enzymatic detection of glucose**

Prabisha K.E^1,2+^, Neena P.K^1,2+^, Menon Ankitha^3^, P Abdul Rasheed^3,4*^, P V Suneesh^1,3^, TG Satheesh Babu ^1,3*^

^1^ Department of Chemistry, Amrita School of Physical Sciences Coimbatore, Amrita Vishwa Vidyapeetham, Coimbatore, 641112, India

^2^ Amrita Biosensor Research Laboratory, Amrita School of Engineering Coimbatore, Amrita Vishwa Vidyapeetham, Coimbatore, 641112, India

^3^Department of Chemistry, Indian Institute of Technology, Palakkad, Kerala, 678 623, India

^4^Department of Biological Sciences and Engineering, Indian Institute of Technology, Palakkad, Kerala, 678623, India

+Equal Author

*Corresponding Author

Corresponding authors Email ID: [tg_satheesh@cb.amrita.edu](mailto:tg_satheesh@cb.amrita.edu) (T G Satheesh Babu) [abdulrasheed@iitpkd.ac.in](mailto:abdulrasheed@iitpkd.ac.in) (P Abdul Rasheed)

Phone number: +919442368632 Fax: 0422-2686274

**Electrochemical characterization of Nb_2_CT_x_@Se/Au towards glucose detection**


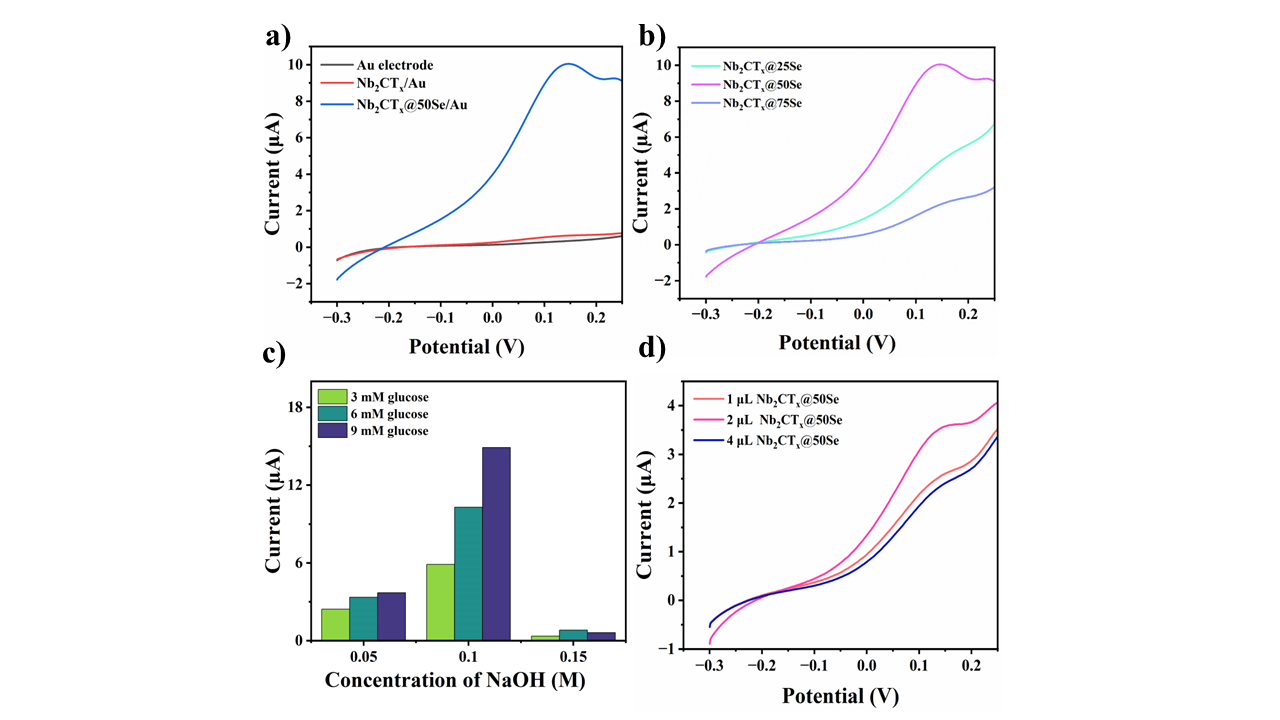


***Fig. S1:*** *LSV response of* ***a)*** *bare Au electrode, Nb_2_CT_x_/Au, and Nb_2_CT_x_@50Se/Au****. b)*** *Nb_2_CT_x_@25Se/Au, Nb_2_CT_x_@50Se/Au, and Nb_2_CT_x_@75Se/Au with 0.1 M NaOH with 6 mM glucose at the scan rate of 0.05 Vs^-1^. Optimization of* ***c)*** *electrolyte (NaOH) concentration* ***d)*** *Effect of material loading onto the electrode towards glucose oxidation.*

**Scan rate study**


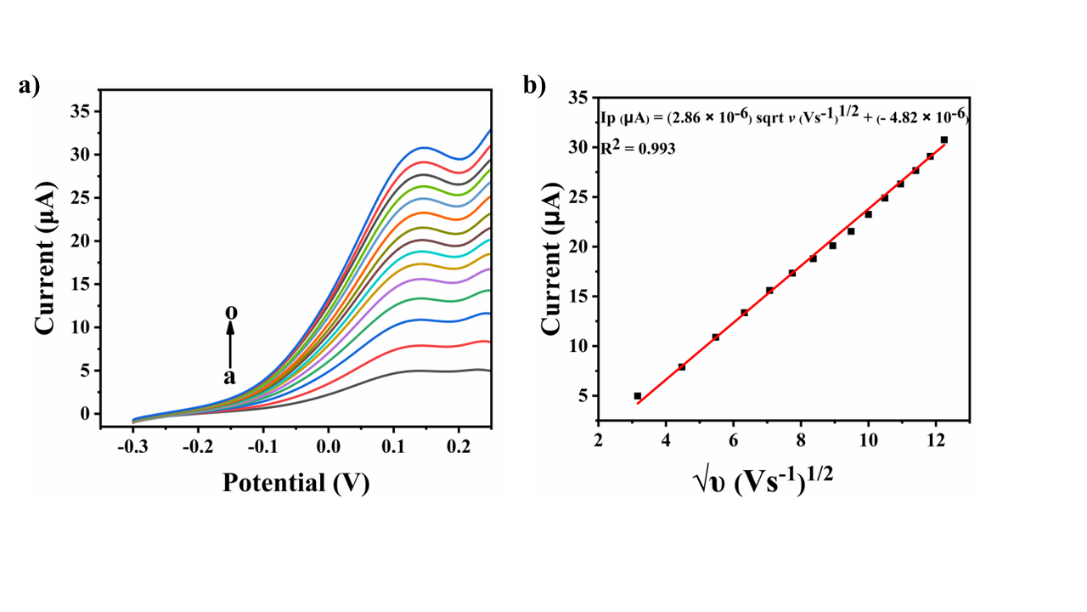


***Fig S2: (a)*** *LSV of Nb_2_CT_x_@50Se/Au in 0.1M NaOH with various scan rates from 0.01 V s^-1^ to 0.15 V s^-1^ (a - o)* ***(b)*** *Linear plot of sqrt. scan rate vs peak current.*

**Selectivity and Reproducibility**


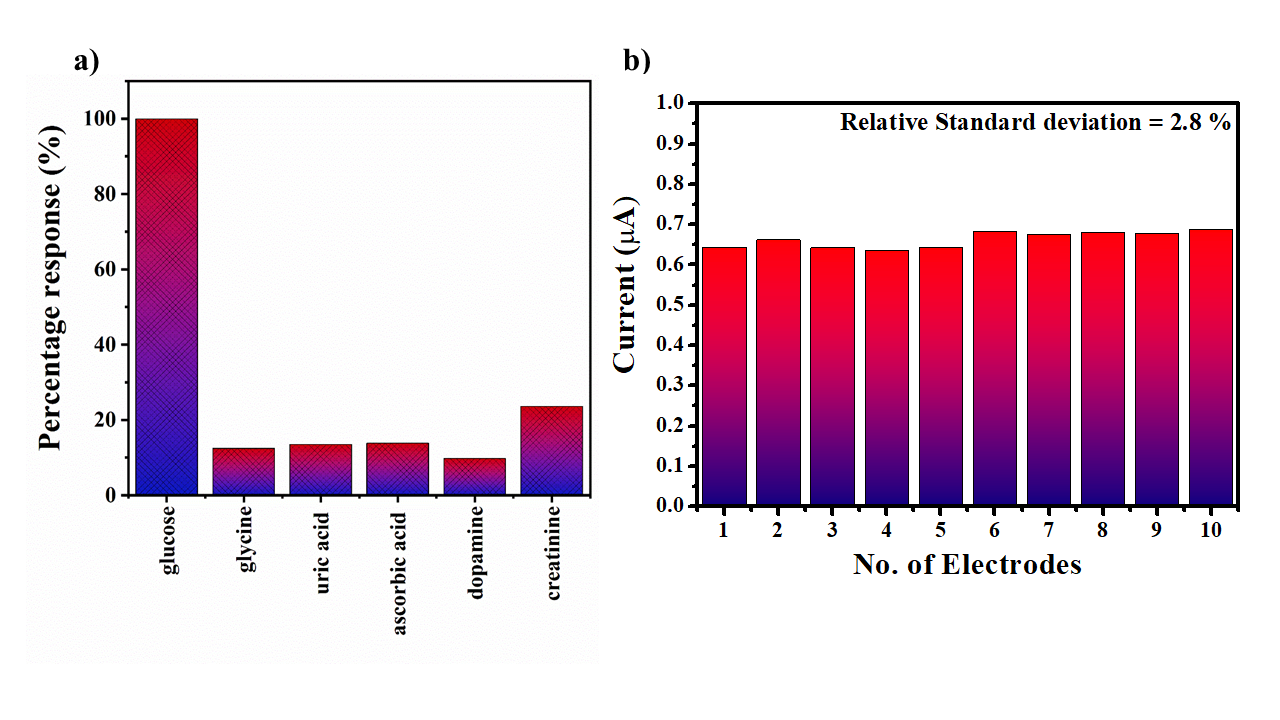


***Fig S3: a)*** *Current response obtained on Nb_2_CTx@50Se/Au with 12 mM glucose in the presence of potentially interfering molecules (100 µM dopamine, 0.25 mM glycine, 10 µM uric acid, 200 µM creatinine and 100 µM ascorbic acid) and* ***b)*** *current response obtained for ten different sensor electrode.*
